# The Effect of GPRC5a on the Proliferation, Migration Ability, Chemotherapy Resistance, and Phosphorylation of GSK-3β in Pancreatic Cancer

**DOI:** 10.3390/ijms19071870

**Published:** 2018-06-26

**Authors:** Bin Liu, Hai Yang, Christian Pilarsky, Georg F. Weber

**Affiliations:** Department of Surgery, Universitätsklinikum Erlangen, Krankenhausstraße 12, 91054 Erlangen, Germany; Liu.Bin@uk-erlangen.de (B.L.); Hai.Yang@uk-erlangen.de (H.Y.); Georg.Weber@uk-erlangen.de (G.F.W.)

**Keywords:** pancreatic cancer, *GPRC5a*, CRISPR/Cas9 system, GSK-3β

## Abstract

Pancreatic cancer (PaCa) is the fourth leading cause of cancer-related death, and personalized targeted cancer therapy is becoming a promising treatment strategy for PaCa. The central approach of targeted therapy is to find a targetable key and an effective targeting method. In this study, the importance of *GPRC5a* (the G-protein-coupled receptor family C, member 5, group A) was identified using data mining methods based on published datasets. After analysis of the basic expression of *GPRC5a* in normal pancreas tissue and various PaCa cell lines, gene editing of *GPRC5a* in the human PaCa cell line MIA PaCa-2 and the mouse PaCa cell line TB32047 was performed using CRISPR/Cas9 (Clustered Regularly Interspaced Short Palindromic Repeats/CRISPR-associated proteins 9) to investigate the influence of *GPRC5a* on the proliferation and migration of PaCa cells as well as its effects on chemotherapy drug resistance. The results showed that *GPRC5a* was upregulated in PaCa tissues and various PaCa cell lines. Knockout of *GPRC5a* reduced the proliferation and migration ability of PaCa cell lines and suppressed the chemotherapy drug resistance of gemcitabine, oxaliplatin, and fluorouracil in PaCa cells. The phosphorylation of GSK-3β (Glycogen synthase kinase-3β) was found to be upregulated in the MIA PaCa-2 and TB32047 cells after *GPRC5a* knockout. In conclusion, *GPRC5a* was upregulated in PaCa leading to an enhanced drug resistance in PaCa cells. These results provide for the first time a theoretical basis for the development of an improved PaCa targeted therapy.

## 1. Introduction

Pancreatic cancer (PaCa) is one of few cancer types showing no improvement over the course of the 5-year survival rate [1]. PaCa was diagnosed in approximately 53,070 patients, with an estimated 41,780 deaths, in the United States in 2016 [2]. In China, the incidence of PaCa was in an upward trend from 2000 to 2011, and PaCa is one of the top 10 most common cancers in both men and women [3]. In Europe, PaCa is currently one of the most lethal cancers, with a 5-year survival of approximately 7%. It is expected to rise to second place behind lung cancer by 2030 [4]. Surgery is currently the only potentially curative treatment for PaCa [5]. However, surgical treatment is subjected to some limitations, such as rapid disease progression, late diagnosis at advanced, unresectable stages, and inadequate response to current drug regimens [6,7]. Personalized targeted cancer therapy is a promising treatment strategy for PaCa. The basic concept of targeted therapy is to find a targetable key in cancer as well as an effective targeting method [8,9]. In this study, we combined data-mining methods and the CRISPR/Cas9 system to research the correlation between one gene—the G-protein-coupled receptor family C, member 5, group A (*GPRC5a*)—and PaCa. We want to investigate the gene’s biology functions in PaCa and its potential targeted value in personalized therapy.

*GPRC5a*, also named retinoic acid-inducible 3 (RAI3), is a family member of the G-protein-coupled receptors, which is the largest protein superfamily with more than 700 genes in the human genome [10]. *GPRC5a* was originally identified in 1998 in a human oral squamous carcinoma cell line. *GPRC5a* is overexpressed in a variety of cancers, including colon cancer, breast cancer, and gastric cancer [11]. A previous study from our group showed higher expression levels of *GPRC5a* in PaCa tissues than in normal tissues [12]. Therefore, we thought to investigate *GPRC5a* as a potential gene of targeted therapy.

In this study, data-mining methods were used as a powerful supplement to prove the importance of *GPRC5a* in PaCa and the expression of *GPRC5a* in various PaCa cell lines was analyzed. We analyzed the potential functions of *GPRC5a* in PaCa with CRISPR/Cas9 system knockout cells and its relevance to chemoresistance.

## 2. Results

### 2.1. The Data-Mining Analysis for GPRC5a in PaCa

**Figure 1:**
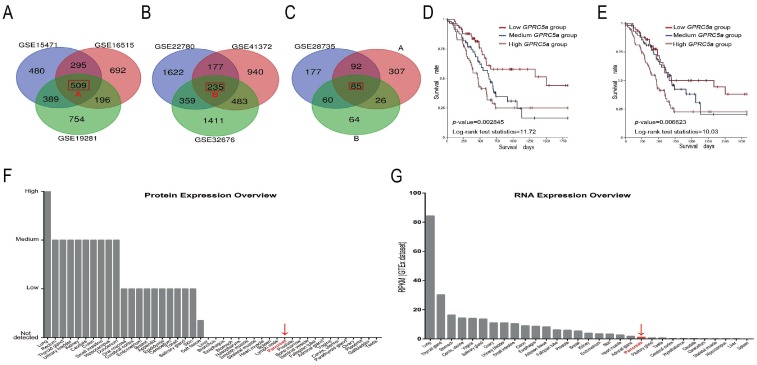
Data-mining analysis of *GPRC5a* in PaCa. (**A**–**C**) Identification of differentially expressed genes (DEGs) in seven mRNA expression-profiling datasets. *GPRC5a* is 1 of the 85 DEGs. The red frame named A means the DEGs among GSE15471, GSE16516 and GSE 19281. The red frame named B means the DEGs among GSE22780, GSE41372 and GSE 32676. The red frame in Figure 1C means the DEGs among GSE28735, red frame A and red frame B. (**D**,**E**) Prognostic value of *GPRC5a* in PaCa patients with different datasets. High mRNA-level expression of *GPRC5a* was associated with worse overall survival in the GDC (Genomic Data Commons) TCGA PaCa (223 samples) (**D**) and the TCGA PaCa (196 samples) datasets (**E**). (**F**,**G**) Basic expression levels of *GPRC5a* in different organs. The protein-level expression of *GPRC5a* was relatively low in the pancreas compared with that in the other 43 organs (**F**), similar to the mRNA expression of *GPRC5a* (**G**).

**Figure 2:**
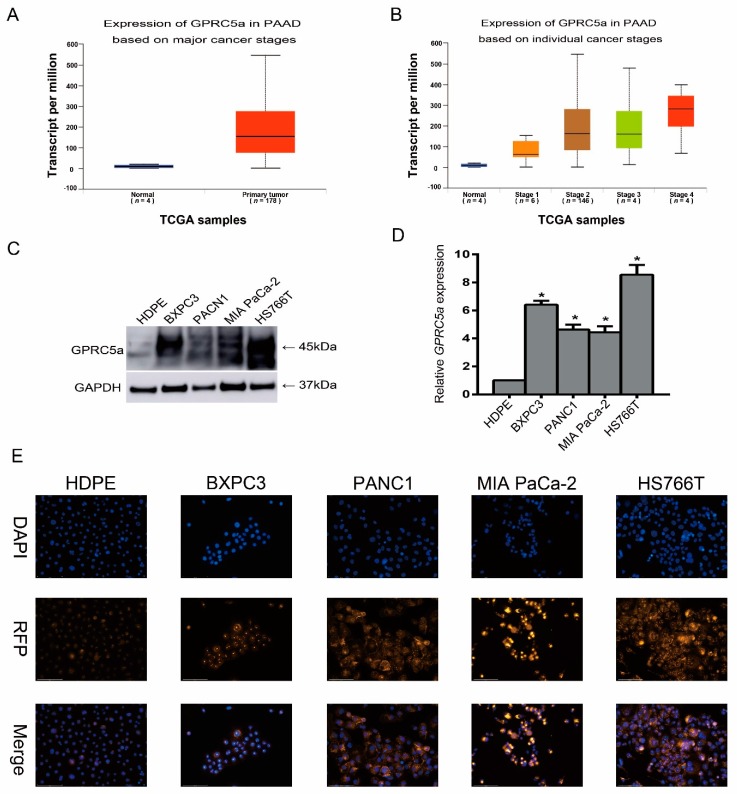
*GPRC5a* expression levels in normal pancreas and PaCa (PAAD) tissue and cell lines. (**A**,**B**) The data-mining analysis showed that *GPRC5a* was significantly upregulated in PaCa (PAAD) samples with an association of increased expression in higher malignant tumor stages (*p* < 0.05). (**C**,**D**) The Western blot and qPCR results showed that the basic protein-level expression of *GPRC5a* in PaCa cell lines was higher than that in the HPDEE6E7 pancreas cell line. (**E**) Immunofluorescence assay showed upregulated expression of *GPRC5a* in PaCa cell lines (200×). The error bars were from multiple samples (*n*) measurement results (**A**,**B**) or three experimental measurements (**C**,**D**). * *p*-value < 0.05.

We identified 1673, 1692, 1848, 2393, 414, 2488, and 1835 differentially expressed genes (DEGs) in PaCa compared to normal tissue in the GSE15471, GSE16515, GSE19281, GSE22780, GSE28735, GSE32676, and GSE41372 datasets. A total of 85 genes were considered DEGs in all datasets (Figure 1A–C) and *GPRC5a* is one of the 85 DEGs (Table 1). Overall survival analysis was conducted to detect the relationship between *GPRC5a* expression and PaCa outcome. High mRNA expression of *GPRC5a* was associated with worse overall survival in both datasets (log-ranks *p*-value = 0.002845 and 0.006623, Figure 1C,D) confirming our previous results [12]. Furthermore, “The Human Protein Atlas” was used to measure the basic expression level of *GPRC5a* in different human organs. The basic mRNA expression level of *GPRC5a* in the normal tissue of the pancreas was lower than that in other organs using the HPA (Human Protein Atlas) dataset, the GTEx (Genotype-Tissue Expression) dataset, and the FANTOM5 (The Functional Annotation of the Mammalian Genome 5) dataset (Figure 1F,G, HPA and FANTOM5 showed the same results). Moreover, the basic expression levels of *GPRC5a* in pancreatic cancer tissues at different stages were analyzed with the TCGA (The Cancer Genome Atlas) dataset. The results showed that the expression level of *GPRC5a* increased with higher stages of PaCa (Figure 2A,B).

### 2.2. Expression of GPRC5a in PaCa Cell Lines

To further investigate the expression of *GPRC5a* in PaCa, we analyzed the protein expression levels in the normal pancreas HDPEE6E7 cell line and different PaCa cell lines by Western blot and immunofluorescence assay. The results showed that the protein expression level of *GPRC5a* in PaCa cell lines was significantly higher as compared to the normal pancreas cell line. Q-PCR analysis similarly showed a higher relative expression of *GPRC5a* in the PaCa cell lines as compared to the normal pancreas cell line (Figure 2C,D). Immunofluorescent analysis showed overexpression of *GPRC5a* in PaCa cell lines as compared to the normal pancreas HDPEE6E7 cell line (Figure 2E).

### 2.3. Inhibition of the Proliferation and Migration Ability of TB32047 and MIA PaCa-2 Cells by Knockout of GPRC5a

**Figure 3:**
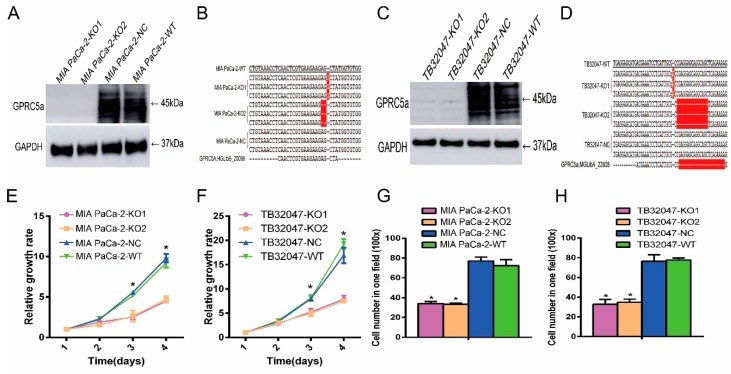
Influence of *GPRC5a* knockout on the proliferation and migration ability in MIA PaCa-2 and TB32047 cells. (**A**–**D**) Western blot and sequencing results showing the *GPRC5a* knocked out in MIA PaCa-2-KO1/2 and TB32047-KO1/2 (Red highlight area means the different part in comparison of sequencing sequence). (**E**,**F**): The knockout of *GPRC5a* inhibited the proliferation ability of MIA PaCa-2 and TB32047 cells. (**G**,**H**) *GPRC5a* knockout inhibited the migration ability of MIA PaCa-2 (**G**) and TB32047 cells (**H**). * *p*-value < 0.05.

We next analyzed *GPRC5a*’s function in the MIA PaCa-2 and KPC (Pdx1-Cre; K-Ras^+/LSLG12D^; p53^R172H/+^) mouse-model-derived TB32047 cell lines. The CRISPR/Cas9 gene editing system was used to knockout *GPRC5a* in those two cell lines. Western blot and sequencing were performed to detect the knockout effect. The results showed no protein expression in knockout MIA PaCa-2 and TB32047 cells (Figure 3A,C). In/del mutations in the sequence of *GPRC5a* were detected in all cells with *GPRC5a* knockout (Figure 3B,D). The proliferation ability of cells with *GPRC5a* knockout was compared with wild-type (WT) and negative control cells. The results showed that cells with *GPRC5a* knockout grew slower as compared to negative control and wild-type cells (Figure 3E,F). Furthermore, the migration ability of cells with *GPRC5a* knockout was detected. The results showed that the migration ability of cells with *GPRC5a* knockout was lower as compared to negative control and wild-type cells (Figure 3G,H).

### 2.4. Inhibition of the Chemotherapy Drug Resistance in TB32047 and MIA PaCa-2 Cells by Knockout of GPRC5a

**Figure 4:**
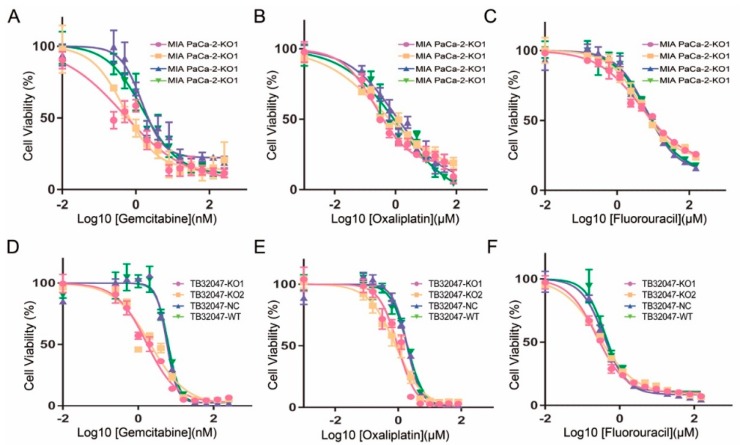
Knockout of *GPRC5a* suppressed chemotherapy drugs resistance in MIA PaCa-2 and TB32047 cells. (**A**–**C**) shows the EC50 (Concentration for 50% of maximal effect) assay of gemcitabine, oxaliplatin, and fluorouracil in the MIA PcCa-2 cell line. (**D**–**F**) shows the same results in the TB32047 cell line.

We analyzed the response to the commonly used drugs 5-fluorouracil, gemcitabine, and oxaliplatin in the *GPRC5a* knockout system. In the *GPRC5a* knockout cell line, we observed a reduced resistance against these drugs compared to the WT cell line or the negative control cell line (Table 2, Figure 4 and Figure S1).

### 2.5. Knockout of GPRC5a Promotes the Phosphorylation of GSK-3β at Ser9 in TB32047 and MIA PaCa-2 Cells

**Figure 5:**
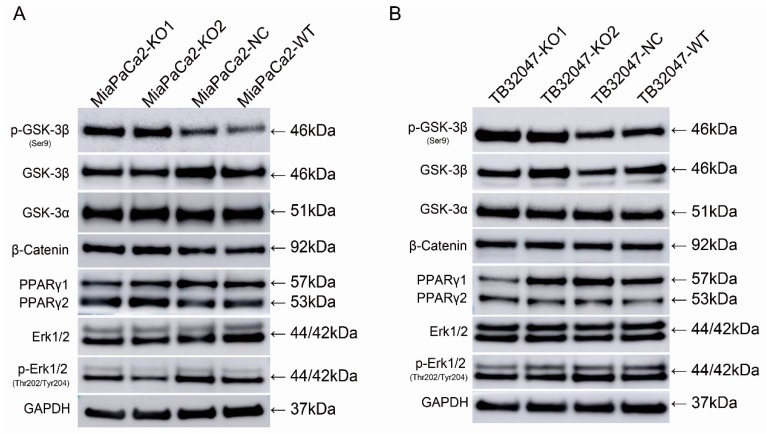
Knockout of *GPRC5a* promotes the phosphorylation of GSK-3β at Ser9. (**A**,**B**) shows GSK-3β and its related upstream and downstream protein expression with *GPRC5a* knockout in MIA PaCa-2 and TB32047, respectively.

The knockout of *GPRC5a* led to an upregulation of the phosphorylation of GSK-3β (Ser9) (Glycogen synthase kinase-3β )in MIA PaCa-2 and TB32047 cells. There was no significant change in the expression of GSK-3β, GSK-3α, β-Catenin, PPARγ (Peroxisome Proliferator-Activated Receptor γ), and Erk1/2 (extracellular signal-regulated kinase 1/2) or in its activated phosphorylation at Thr202/Tyr204 (Figure 5 and Figure S2).

## 3. Discussion

G-protein-coupled receptors are the largest protein superfamily with more than 700 genes in the human genome [10] playing an important role in a variety of biological processes [13]. This protein superfamily also acts as a drug target in many different diseases and more than 40% of FDA (Food and Drug Administration)-approved drugs target GPCRs (G Protein-Coupled Receptors) or GPCR-associated pathways [14]. GPCRs also play an integral role in regulating and activating cancer-associated signaling pathways; therefore, they may be used as biomarkers for the early diagnosis of various cancers [15]. Further investigation of the pharmacological potential of GPCRs and their downstream regulators is required in order to develop therapies that can efficiently target cell signaling pathways in cancer [16,17].

*GPRC5a*, also named *RAI3* (Retinoic acid-induced protein 3), located on chromosome 12p13–p12.3, has been found to play significant roles in some biological processes, such as cell proliferation and the cell cycle. However, the influences of *GPRC5a* on different cancers vary. *GPRC5a* was reported to be strongly expressed in the lung [18,19] and is regarded as a tumor suppressor in lung cancer as well as in head and neck squamous cell carcinoma [20]. However, there are many papers that have reported that the higher expression of *GPRC5a* correlated with a worse survival rate in colon cancer, breast cancer, and gastric cancer [11]. In our previous study, *GPRC5a* was reported to have lower expression levels in normal pancreas tissues and have a key influence on the proliferation of PaCa cells via STAT3 (Signal transducer and activator of transcription 3)-modulated pathways [12].

Data mining is a process of discovering patterns in large data sets and involves methods at the intersection of machine learning, statistics, and databases. It has been used in many biological applications [21,22]. The CRISPR/Cas9 gene editing system is a new technology to knock out genes at the DNA level [23]. As compared to RNAi, the pre-transcriptional knockout improves the functional analysis of a gene expression loss. In this study, data mining and CRISPR/Cas9 gene editing were used to investigate the role of *GPRC5a* from PaCa cell lines MIA PaCa-2 and TB32047.

Our data-mining approach confirmed previous results that *GPRC5a* is overexpressed in PaCa tissue and that this is associated with a shorter overall survival time [12].

It indicates that lowering the expression of *GPRC5a* may improve the prognosis and suggests that *GPRC5a* is an important gene in PaCa development. We then performed *GPRC5a* knockout experiments to elucidate the role of *GPRC5a* in PaCa.

Therefore, we analyzed the basic expression of *GPRC5a* in the normal pancreas cell line HPDEE6E7 and PaCa cell lines. The result showed that *GPRC5a* was upregulated in all PaCa cell lines at both the protein and mRNA levels. In order to analyze the function of *GPRC5a* in PaCa, *GPRC5a* was knocked out from the MIA PaCa-2 and TB32047 cell lines with the CRISPR/Cas9 gene editing system, the knockout effect was evaluated by Western blot and sequencing, and the influence of *GPRC5a* on proliferation and migration ability was analyzed. The results showed that cells with *GPRC5a* knockout grew or migrated slower than negative control and wild-type cells, indicating that the knockout of *GPRC5a* inhibited proliferation and migration ability in PaCa cell lines.

Secondly, we detected the drug resistance of chemotherapy after knockout of *GPRC5a* from the MIA PaCa-2 and TB32047 cell lines by EC50 assay. The results showed that knocking out *GPRC5a* from PaCa can suppress its resistance of the chemotherapy drugs gemcitabine, oxaliplatin, and fluorouracil. Gemcitabine is a synthetic pyrimidine nucleoside prodrug, which has been used in various carcinomas, including PaCa [24]. Oxaliplatin has also been used as chemotherapy drug in a variety of carcinomas, and it is worth noting that oxaliplatin is always used in combination with Fluorouracil [25]. Their inhibitory effect on PaCa has been proven in both experimental research and clinical treatment. However, all of these drugs have been reported to have side effects, which greatly limit their therapeutic applications. In this study, the drug sensitivity for gemcitabine, oxaliplatin, and fluorouracil by the knockout of *GPRC5a* could be increased, meaning that similar chemotherapy effects with lower concentrations of chemotherapy could be obtained in PaCa cells. As a consequence, the side effects may be significantly reduced if a *GPRC5a* knockout from PaCa patients’ cancer cells is performed. This may be a potential targeted therapy, especially for PaCa patients with chemotherapy resistance. Interestingly, the inhibitory effects of the *GPRC5a* knockout were different in the three investigated drugs, whereby the sensitivity effect for Fluorouracil was smaller than that for the other two drugs.

It has been reported that the Gemcitabine resistance in PaCa has a close relationship with Akt-GSK3β-Snail pathway activity, and it has been shown that oxaliplatin induces an epithelial–mesenchymal transition in cancers. Moreover, two main pathways, including GSK3β and PPARγ as hub genes, for the anti-cancer function of Fluorouracil in PaCa have been previously described [26,27]. In this study, we found that the activity of GSK3β was downregulated in cells with *GPRC5a* knockout, but no significant change in the expression of PPARγ could be observed. The PPARγ-related pathways may have compensatory effects for the *GPRC5a*-suppressed Fluorouracil resistance. Of course, further in-depth studies are still needed on this issue.

GSK3 is a family of serine/threonine protein kinases comprising two highly conserved isoforms, GSK3α and GSK3β, which show approximately 85% overall homology and 98% homology in their kinase domains, respectively [28]. Of the two GSK3 isoforms, GSK3β has been studied most as the functional redundancy of the two isoforms in regulating the canonical Wnt/β-Catenin pathway [26,29]. In PaCa, it has been reported that inhibiting GSK3β activity with inhibitors or RNAi can preferentially attenuate the survival and proliferation of tumor cells and induce them to undergo apoptosis [30,31]. GSK3β also has a close relationship with therapy resistance [26]. Others showed that a GSK3β-specific pharmacological inhibitor, named AR-A014418, given in combination with chemotherapeutic agents, had additive and synergistic effects [32]. Many studies show that GSK3β participates in chemotherapy, radiotherapy, and targeted therapies via multiple molecular pathways [33]. In this study, we found that p-GSK-3β at Ser9, the inactivated state of GSK3β, was upregulated in cells with *GPRC5a* knockout. However, the total expression amount of GSK3β and GSK3α remained unchanged. This shows that the phosphorylation state of GSK-3β has an inner relationship with *GPRC5a* expression. In addition, we detected the upstream- and downstream-related proteins in the main GSK-3β pathway. β-Catenin is the main downstream gene in the Wnt/GSK-3β/β-Catenin pathway. The unchanged expression of β-Catenin indicates that *GPRC5a* has little influence on this pathway. Erk1/2 is the upstream gene of the Erk/GSK-3β pathway. In our previous study, Erk1/2 and p-Erk1/2 were not regulated with *GPRC5a* by RNAi interference. In this study, we confirmed these results that *GPRC5a* cannot regulate Erk1/2 or its phosphorylation state. The results suggested that *GPRC5a* may affect GSK-3β activity by promoting the GSK-3β inactivity phosphorylation state at Ser9. However, further investigations need to be conducted in the future to understand the role of *GPRC5a* in the regulation of GSK-3β activity.

## 4. Methods

### 4.1. Data-Mining Analysis

An Expression Omnibus database (GEO) dataset, in which a comparison between normal and PaCa tissue was performed, was identified (GSE16515 [34], GSE15471 [35], GSE28735 [36], GSE19281 [37], GSE22780 [38], GSE32676 [39], and GSE41372 [40]).

The seven gene expression datasets include 166 cancer tissue samples and 127 normal tissue samples from PaCa patients. All these datasets were generated on Affymetrix chips and uploaded by independent groups.

GEO2R is an online tool (https://www.ncbi.nlm.nih.gov/geo/geo2r) which can compare different groups in a GEO series. In this study, GEO2R was used to identify differentially expressed genes (DEGs) between PaCa and normal tissue samples. The PaCa samples were regarded as the “cancer group” while the normal samples were regarded as the “control group”. The genes for which the adjusted (Benjamini and Hochberg or False discovery rate) *p*-value was smaller than 0.01 and the absolute log fold-change was greater than 1 were selected.

The online tool “Calculate and draw custom Venn diagrams” (http://bioinformatics.psb.ugent.be/webtools/Venn/) was employed to identify genes that were up or downregulated in all seven GEO datasets. As this tool is not able to deal with more than six groups in one time, the first three groups of gene expression datasets were selected, and then the remaining three groups of datasets were selected. Then, the other one group and the two above collection groups of datasets were analyzed together. After that, all DEGs were analyzed, and the expression level of *GPRC5a* in those seven gene expression microarrays was detected.

The UCSC Xena (https://xenabrowser.net/) was adopted to perform overall survival analysis in PaCa. In this study, the TCGA Pancreatic Cancer (PAAD) and GDC TCGA Pancreatic Cancer datasets were selected to create Kaplan–Meier plots based on the gene expression level of *GPRC5a*. Hazard ratios with 95% confidence intervals and Log-rank *p*-values were recorded. The top one-third, middle one-third, and bottom one-third percentiles of *GPRC5a* expression values were considered to be the high, middle, and low groups, respectively. Then, the relationship between *GPRC5a* expression and survival time was detected.

Furthermore, the basic expression level of *GPRC5a* in different human organs was measured by “The Human Protein Atlas v17” [41]. There were three datasets in the website, including the HPA dataset (RNA-seq tissue data is reported as mean transcripts per million, corresponding to the mean values of the different individual samples from each tissue), the GTEx dataset (RNA-seq data is reported as median reads per kilobase per million mapped reads, generated by the Genotype-Tissue Expression project), and the FANTOM5 dataset (tissue data obtained by Cap Analysis of Gene Expression is reported as Tags Per Million, generated by the FANTOM5 project). The three datasets were combined and used in this study. In addition, the different expressions of *GPRC5a* between normal pancreas and PaCa tissues were measured. UALCAN is an easy online tool (http://ualcan.path.uab.edu) for in-depth analyses of TCGA gene expression data [42]. It uses TCGA level 3 RNA-seq and clinical data from 31 cancer types. In this study, UALCAN was used to identify the up or downregulation of *GPRC5a* in PaCa.

### 4.2. Cell Culture

In this study, the human PaCa lines BxPC3, PANC1, HS766T, and MIA PaCa-2 and the normal pancreas cell line HDPEE6E7 were used. BXPC3, PANC1, HS766T, and MIA PaCa-2 were purchased from ATCC (American Type Culture Collection, Manassas, VA, USA). HDPEE6E7 was a gift from Ming-Sound Tsao, UHN (University Health Network), Toronto, ON, Canada. Primary mouse PaCa cell line TB32047 was provided by David Tuveson, CSHL (Cold Spring Harbor Laboratory, Cold Spring Harbor, NY, USA). All cell lines were grown in monolayer culture in a humidified atmosphere containing 5% CO_2_ at 37 °C. The culture medium for BXPC3 and PANC1 consisted of RPMI (Roswell Park Memorial Institute) Medium 1640 (Gibco, 21875-034, Waltham, MA, USA) with 10% fetal bovine serum (FBS) (Gibco, A31608-01). HS766T and TB32047 cells were cultured in DMEM (Dulbecco’s Modified Eagle’s medium) medium (Gibco, 30966-021) with 10% FBS. MIA PaCa-2 cells were cultured in DMEM medium with 10% FBS and 2% horse serum (Gibco, 16050-130). HDPEE6E7 cells were cultured in Keratinocyte-SFM (Keratinocyte-serum-free medium) medium (Gibco, 10724-011). All cells were harvested by 0.25% Trypsin-EDTA (Ethylenediaminetetraacetic acid) (Gibco, 25200-072).

### 4.3. Western Blot

Cells were lysed with RIPA-buffer (Radioimmunoprecipitation assay buffer). Gel electrophoresis was performed under reducing conditions with acrylamide gel and proteins were transferred to a nitrocellulose membrane. As primary antibodies, GPRC5a (NOVUS, NBP1-89743, Littleton, CO, USA), phosphorylation -GSK-3β (Cell Signaling, #5558, Danvers, MA, USA ), GSK-3β (Cell Signaling, #12456), GSK-3α (Cell Signaling, #4337), β-Catenin (Cell Signaling, #8480), PPARγ (Cell Signaling, #2443), Erk1/2 (Cell Signaling, #4695), and phosphorylation Erk1/2 (Cell Signaling, #4370) were used for detection. GAPDH (Cell Signaling Technology, 5175S) served as loading control. HRP (Horseradish peroxidase) -linked anti-rabbit IgG (Immunoglobulin G) (Cell Signaling Technology, 7074) was used as the secondary antibody. Quantification of signals was performed by an Amersham Imager 600 (Pittsburgh, PA, USA) with SignalFire Elite ECL Reagent (Cell Signaling Technology, 12757S). All Western blot assays were carried out individually two times, which validates the reliability of the results.

### 4.4. Quantitative RT-PCR

The isolation of RNA was performed with a NucleoSpin RNA Plus kit (Macherey-Nagel, #740984.250, Düren, Nordrhein-Westfalen, Germany) and the cDNA was synthesized with a High Capacity cDNA Reverse Transcription Kit (Applied Biosystems, 00364942, Foster City, CA, USA). Quantitative RT-PCR was conducted with PowerUP SYBR Green Master Mix (Applied Biosystems, A25741) and analyzed by the 2^−ΔΔCt^ method. *GAPDH* was used as a reference value. Primer sequences for amplification were *GPRC5a*-forward (5′-GCTGCTCACAAAGCAACGAA-3′), *GPRC5a*-reverse (5′-ATAGAGCGTGTCCCCTGTCT-3′), *GAPDH*-forward (5′-CTTTGGTATCGTGGAAGGACTC-3′), and *GAPDH*-reverse (5′-AGTAGAGGCAGGGATGATGT-3′). All primers were synthesized by Eurofins Genomics (Ebersberg, Germany).

### 4.5. The CRISPR/Cas9 Gene Editing Knockout System

In this study, *GPRC5a* was knocked out by the CRISPR/Cas9 gene editing system in MIA PaCa-2 and TB32047. pSpCas9(BB)-2A-GFP (PX458, addgene Plasmid #48138) was used as a plasmid vector [43]. *GPRC5a*-human-sgRNA-forward (5′-caccCAACTCGTGAAGAAGAGCTA-3′), *GPRC5a*-human-sgRNA-reverse (5′-aaacTAGCTCTTCTTCACGAGTTG-3′), *GPRC5a*-mouse-sgRNA-forward (5′-caccACGAAATCCTCATTGCGCCG-3′), *GPRC5a*-mouse-sgRNA-reverse (5′-aaacCGGCGCAATGAGGATTTCGT-3′), NC-human-sgRNA-forward (5′-caccATCGTATCATCAGCTAGCGC-3′), NC-human-sgRNA-reverse (5′-aaacGCGCTAGCTGATGATACGAT-3′), NC-mouse-sgRNA-forward (5′-caccGACCGGCCAACGGTAGCGGC-3′), and NC-mouse-sgRNA-reverse (5′-aaacGCCGCTACCGTTGGCCGGTC-3′) were synthesized by Eurofins. The sgRNAs were designed based on the human and mouse CRISPR Knockout Pooled Library (GeCKO v2, Genome-scale CRISPR Knock-Out Version 2, http://genome-engineering.org/) [44]. Plasmid construction was done according to protocol and confirmed by sequencing. MIA PaCa-2 and TB32047 cells were transfected with *GPRC5a* knockout plasmid by Lipofectamine 3000 Transfection Reagent (Invitrogen, #L3000015, Waltham, MA, USA) for 72 h. Then, all the GFP (Green fluorescent protein)-positive cells were sorted by a fluorescence-activated cell sorter. Sixty GFP-positive cells were plated in one 96-well plate for selecting single clones. After a few days of culture, Western blot and sequencing were performed to detect the knockout effect. Sequencing was done by Eurofins, and all selected clones were sequenced at least five times. In this study, we set two of the positive single clones as “KO1” and “KO2” for each cell line. The cell transfected with NC-sgRNA was used as one of the control groups, which was named “NC”. The other control group used wild-type cells and was named “WT”.

### 4.6. Proliferation Assay

We used the cell-counting method to detect the proliferation ability of *GPRC5a* knockout cells and control cells. This method is achieved by fluorescence microscopy counting the number of cells in each well. Compared with a sampling count, it can count every cell in one well to avoid sampling bias. Cells were plated as 3 × 10^3^ cells per well in grown medium on 96-well black plates (Corning, #3603, Kaiserslautern, Germany) for four days. After 24, 48, and 72 h of proliferation, cells were stained with DAPI by NucBlue Live Cell Stain ReadyProbes reagent (Invitrogen, R37605) and imaged in 38 fields for each well by an Evos FL Auto 2 imaging system (Invitrogen, AMAFD2000). Images were counted by HCS studio cell analysis software V2.0 (Thermo, SX000041A, Waltham, MA, USA). All experiments were performed in duplicate and repeated at least three times.

### 4.7. Migration Assay

A FluoroBlok^TM^ Insert system (Corning, REF351152, Kaiserslautern Germany) was used to analyze the migration ability of *GPRC5a* knockout clones and the negative control clone. After being cultured for 24 h with free FCS medium, cells were plated as 25,000 cells per well in a FluoroBlok^TM^ Insert, and the FluoroBlok^TM^ Insert was put into a Multiwell 24 Well (Corning, REF353504). The medium in the up-well was free FCS medium and that in the down-well was grow medium with an extra 10% FCS. After 16 h of migration in an incubator, NucSpot Live 488 Nuclear Stain (Biotium, Cat40081, Fremont, CA, USA) was used. The cells, attached on the center of the membrane, were imaged in a random four fields for each well by the Evos FL Auto 2 imaging system (Invitrogen, AMAFD2000). The positive points in all images were counted manually.

### 4.8. Chemotherapy Drugs Resistance Assay

The influence of *GPRC5a* on PaCa was detected by the EC50 method. Cells were plated as 3 × 10^3^ cells per well in grown medium on a 96-well black plate for 24 h. Then, the chemotherapy drug was added to the well to final concentration. After cells were cultured for another 72 h, they were counted with the same method used in the above proliferation assay. EC50 was calculated and plotted by GraphPad Version 7 (GraphPad Software, La Jolla, CA, USA). Gemcitabine, oxaliplatin, and fluorouracil were used for the EC50 assay in this study. These drugs were purchased from the pharmacy of the Universitätsklinikum Erlangen in ready-made solutions (Gemcitabine, 40 mg/mL, Hexal AG, Holzkirchen, Freistaat Bayern, Germany), (oxaliplatin, 50 mg/mL, Medac GmbH, Wedel, Niedersachsen, Germany), and (Fluorouracil, 5 mg/mL, Accord Medical Ltd., London, UK). Except for the initial concentration, there were 11 gradient concentrations for each drug. The initial concentration was set to 0.01 nM (gemcitabine), 0.01 µM (oxaliplatin), and 0.01 µM (fluorouracil). The gradient concentration range was (0.25–256 nM) for gemcitabine, (0.02–20 µM) for oxaliplatin, and (0.15–153.6 µM) for fluorouracil. The concentration was doubly increased.

### 4.9. Immunofluorescence Assay

Cells were plated in 48-well plates and cultured to an appropriate density. After trypsinization, the cells were covered with 100 µL of 4% warm formaldehyde. The cells were cultured for 15 min at room temperature. Wells were washed with PBS three times. Firstly, block buffer was added, and the cells were cultured for 60 min at room temperature. Then, 100 µL of antibody buffer with 0.5% primary antibody *GPRC5a* (NOVUS, NBP1-89743) were added and the cells were cultured at 4 °C overnight. On the second day, the wells were washed with PBS three times before adding 100 μL of antibody buffer with 0.5% secondary antibody Anti-rabbit IgG (Cell Signaling Technology, 4413S). After that, the wells were placed away from light and cultured for 2 h at room temperature. Then, the secondary antibody was washed away with PBS three times. Finally, the fluorescence assay was carried out by the Evos FL Auto 2 imaging system.

### 4.10. Statistical Analysis

Statistical analysis was performed with GraphPad Version 7 and Microsoft Excel Version 2017 (Microsoft, Seattle, WA, USA). A *t*-test was used in cell culture experiments. *p*-values < 0.05 were considered statistically significant. The EC assay was calculated by GraphPad. After transforming concentrations to logarithms (log10), a log(inhibitor) versus response variable slope (four parameters) was used to calculate the EC50 and 95% CI. All assays were carried out individually two times at least (*n* ≥ 2).

## 5. Conclusions

In conclusion, *GPRC5a* is overexpressed in PaCa tissue and PaCa cell lines. The upregulation of *GPRC5a* in PaCa may be associated with a worse prognosis. Knockout of *GPRC5a* by the CRISPR/Cas9 system can suppress proliferation and migration ability and may promote chemotherapy drug sensitivity in PaCa cells. GSK-3β activity was inhibited by upregulation of phosphorylation GSK-3β at Ser9 in cells with *GPRC5a* knockout. This study provides a reference for developing a targeted therapy for PaCa.

## Figures and Tables

**Table 1:** The expression levels of *GPRC5a* in seven microarray datasets. Compared with normal pancreas tissues, the expression level of *GPRC5a* was upregulated in pancreatic cancer tissues (*p*-value < 0.05 and logFC < −1).

Microarray	*p*-Value	Student’s *t*-Value	logFC
GSE15471	1.12 × 10^−12^	8.689697	2.5051499
GSE16515	1.08 × 10^−10^	8.048017	4.3196152
GSE19281	5.56 × 10^−3^	3.6910787	2.28094556
GSE22780	0.0186679	2.584605	2.44569319
GSE28735	2.89 × 10^−12^	8.059183	1.1751238
GSE32676	1.68 × 10^−3^	3.45	2.23
GSE41372	2.34 × 10^−6^	7.93	3.35

**Table 2:** The EC50 values and 95% CI (Confidence Interval) of chemotherapy drugs in cells.

Cells	Gemcitabine (nM)		Oxaliplatin (µM)		Fluorouracil (µM)	
	EC50	95% CI	EC50	95% CI	EC50	95% CI
MIA PaCa-2-KO1	0.455	0.305–0.605	0.224	0.169–0.300	4.642	3.577–6.340
MIA PaCa-2-KO2	0.476	0.322–0.665	0.357	0.210–0.661	4.502	3.720–5.525
MIA PaCa-2-NC	1.458	1.128–1.938	1.015	0.628–2.011	6.675	5.406–8.424
MIA PaCa-2-WT	1.546	1.122–2.146	1.171	0.614–4.312	6.777	5.321–9.061
TB32047-KO1	0.240	0.212–0.268	1.661	1.378–2.000	0.962	0.772–1.179
TB32047-KO2	0.274	0.242–0.306	2.084	1.487–2.931	0.828	0.666–1.024
TB32047-NC	0.384	0.345–4.266	5.774	5.309–6.281	1.894	1.713–2.092
TB32047-WT	0.413	0.353–0.486	6.178	5.589–6.830	1.947	1.743–2.168

The EC50 values of Gemcitabine and Oxaliplatin in cells with GPRC5a knockout accounted for only 30–40% of that in wild-type (WT) cells. The EC50 values of Fluorouracil in cells with GPRC5a knockout accounted for 50–75% of that in negative control (NC) and WT cells. Values keep three decimal places.

## Supplementary Materials

The following are available online at http://www.mdpi.com/1422-0067/19/7/1870/s1.
