# Addendum: Liu, B. et al. The Effect of GPRC5a on the Proliferation, Migration Ability, Chemotherapy Resistance, and Phosphorylation of GSK-3β in Pancreatic Cancer. *Int. J. Mol. Sci.* 2018, *19*, 1870

**DOI:** 10.3390/ijms20071540

**Published:** 2019-03-27

**Authors:** Bin Liu, Hai Yang, Christian Pilarsky, Georg F. Weber

**Affiliations:** Department of Surgery, Universitätsklinikum Erlangen, Krankenhausstraße 12, 91054, Erlangen, Germany; Liu.Bin@uk-erlangen.de (B.L.); Hai.Yang@uk-erlangen.de (H.Y.); Georg.Weber@uk-erlangen.de (G.F.W.)

It has been brought to our attention that one note was missing in the Acknowledgements section of our published paper [1]. It is important for Bin Liu to apply the degree “Dr. rer. biol. hum.” Therefore, we would like to add this and report the Acknowledgements as follows:

**Acknowledgments:** We would like to thank all of the scientists who were able to put their software and data online. Without the efforts of the research community, the data mining part of this study would have been impossible. The present work was performed in fulfillment of the requirements for obtaining the degree “Dr. rer. biol. hum.”

This addendum does not cause any changes to the results and conclusions in the original published paper.
