# Supplementary material for: The Effect of GPRC5a on the Proliferation, Migration Ability, Chemotherapy Resistance, and Phosphorylation of GSK-3β in Pancreatic Cancer
Source: Int J Mol Sci. 2018 Jun 26;19(7):1870. doi: 10.3390/ijms19071870 (PMC6073545; doi:10.3390/ijms19071870)
Supplement: Supplementary file 1 [file ijms-19-01870-s001.pdf]

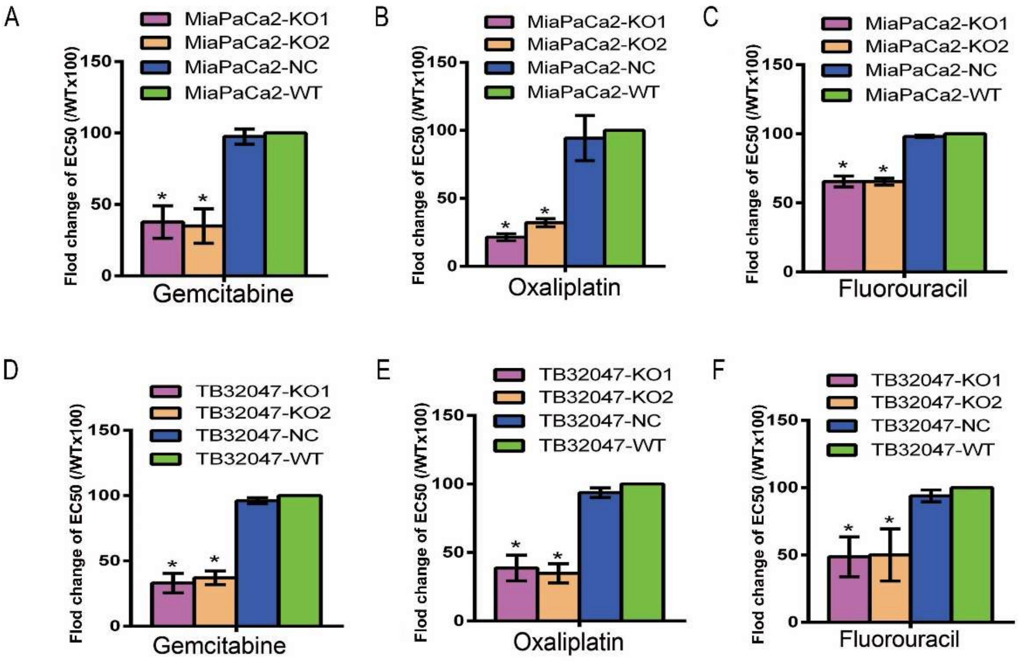

Figure S1. Knockout of GPRC5a suppressed chemotherapy drugs resistance in MIA PaCa-2 and TB32047 cells. (**A**) to (**F**) showed the statistical results of three independent experiments results of IC50 assay in MIA PaCa-2 and TB32047 cells. * P value < 0.05.

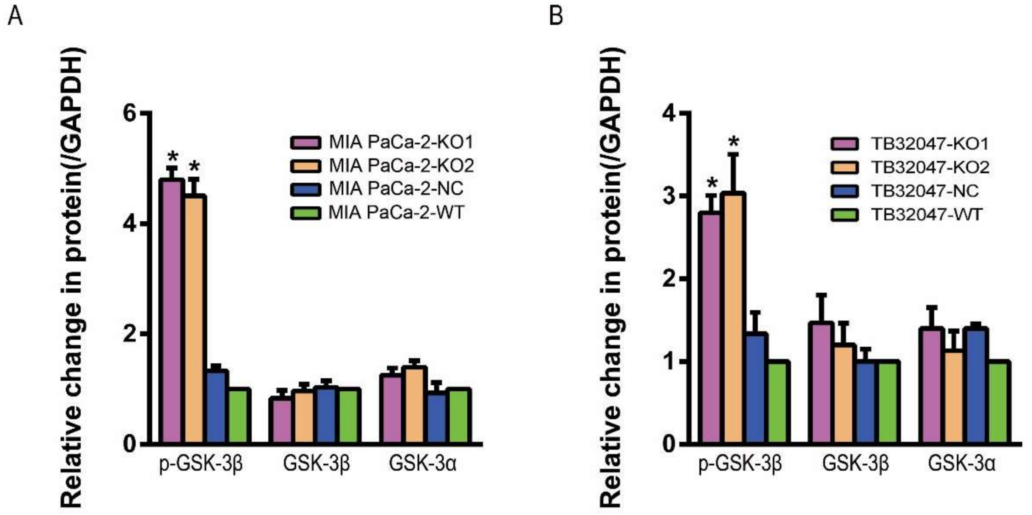

Figure S2. Knockout of GPRC5a promoted phosphorylation GSK-3β at Ser9. (**A**) and (**B**). The three independent experiments western blot results of GSK3 related proteins were quantified by densitometry in MIA PaCa-2 and TB32047 cells. * P value < 0.05.
